# Changes in, and factors associated with, frequency of sex in Britain: evidence from three National Surveys of Sexual Attitudes and Lifestyles (Natsal)

**DOI:** 10.1136/bmj.l1525

**Published:** 2019-05-07

**Authors:** Kaye Wellings, Melissa J Palmer, Kazuyo Machiyama, Emma Slaymaker

**Affiliations:** 1 Faculty of Public Health and Policy, London School of Hygiene and Tropical Medicine, 15-17 Tavistock Place, London WC1H 9SH, UK; 2 Faculty of Epidemiology and Population Health, London School of Hygiene and Tropical Medicine, London, UK

## Abstract

**Objectives:**

To examine changes over time in the reported frequency of occurrence of sex and associations between sexual frequency and selected variables.

**Design:**

Repeat, cross sectional, population based National Surveys of Sexual Attitudes and Lifestyles (Natsal-1, Natsal-2, and Natsal-3).

**Setting:**

British general population.

**Participants:**

18 876 men and women aged 16-59 and resident in Britain were interviewed in Natsal-1, completed in 1991; 11 161 aged 16-44 years in Natsal-2, completed in 2001, and 15 162 aged 16-74 years in Natsal-3, completed in 2012. Comparisons of actual and preferred sexual frequency in men and women aged 16-44 (the age range common to all surveys) between the three surveys. Factors associated with sexual frequency of at least once a week were examined using Natsal-3 data.

**Main outcome measures:**

Sexual activity in the past month; frequency of sex in the past month; preferred frequency of sex.

**Results:**

Median number of occasions of sex in the past month was four in Natsal-1 and Natsal-2 and three in Natsal-3 among women; and three in Natsal-1, Natsal-2, and Natsal-3 among men. The proportion reporting no sex in the past month fell between Natsal-1 and Natsal-2 (from 28.5% to 23.0% in women and from 30.9% to 26.0% in men) but increased significantly in Natsal-3 (to 29.3% in women and 29.2% in men). The proportion reporting sex 10 times or more in the past month increased between Natsal-1 and Natsal-2, from 18.4% to 20.6% in women and from 19.9% to 20.2% in men, but fell in Natsal-3, to 13.2% in woman and 14.4% in men. Participants aged 25 and over, and those married or cohabiting, experienced the steepest declines in sexual frequency (P values for interaction <0.05). Alongside the declines in sexual frequency, there was an increase in the proportion reporting that they would prefer sex more often. Age adjusted odds ratios showed that men and women in better physical and mental health had sex more frequently, as did those who were fully employed and those with higher earnings.

**Conclusions:**

Frequency of sex has declined recently in Britain, more markedly among those in early middle age and those who are married or cohabiting. The findings and their implications need to be explained in the context of technological, demographic, and social change in Britain and warrant further investigation.

## Introduction

Several high income countries have recently reported a decline in the frequency with which men and women have sex.^1^ ^2^ ^3^ ^4^ ^5^ Sexual inactivity might not seem an obvious focus for public health attention—concern is generally reserved for sexual activity and its adverse outcomes such as unintended pregnancy, sexually transmitted infection, and sexual dysfunction—but regular sexual activity has benefits for health, wellbeing, and quality of life. Research indicates that men and women who enjoy an active sex life are fitter, happier,^6^ ^7^ and have better cognitive function^8^ and increased life expectancy.^9^ Evidence shows that sexual activity might help prevent infection by bolstering immune function^10^ ^11^; protect against cardiovascular disease by lowering heart rate and blood pressure;^12^ and reduce stress by increasing release of oxytocin.^13^

These findings should be interpreted cautiously, particularly those that are cross sectional, because of possible confounding and reverse causality. Sexual activity might enhance health, but the converse is also true—men and women in better health are likely to be more sexually active.^14^ ^15^ Nevertheless, the UK NHS considers the evidence to be sufficiently convincing to recommend sexual activity for its health enhancing effects, with the claim, “Weekly sex might help fend off illness.”^16^

We use data from 16 to 44 year old participants in three successive waves of the British National Survey of Sexual Attitudes and Lifestyles (Natsal-1, Natsal-2, and Natsal-3) to measure changes in frequency (actual and preferred) of sex over time and in different sociodemographic groups and to examine factors associated with sexual frequency.

## Methods

### Participants and procedures

Natsal-1, Natsal-2, and Natsal-3 are stratified probability sample surveys of men and women resident in Britain, completed in 1991, 2001, and 2012 (see supplementary table). In Natsal-1, 18 876 people aged 16-59 years were interviewed, 13 765 of whom were aged 16-44.^17^ Natsal-2 comprised 11 161 participants aged 16-44 years,^18^ and Natsal-3 comprised 15 162 participants aged 16-74 years, 9902 of whom were aged 16-44.^19^ ^20^ In all three surveys, stratified random probability sampling was used to select households, from which one eligible individual was randomly selected and invited to participate. The overall response rate was 66.8% for Natsal-1, 65.4% for Natsal-2, and 57.7% for Natsal-3.

Similar measures and procedures were used for all three surveys, and the variables compared in this study were derived from identically worded questions. In Natsal-1, pen and paper were used for the face-to-face and self completed interviews and for the self completed booklet of more sensitive questions. In Natsal-2 and Natsal-3, all participants were interviewed using computer assisted face-to-face and self interviews. Full details of the methods and demographic characteristics of participants are reported elsewhere.^21^

### Measures

Our measures of sexual frequency were derived from questions in the computer assisted self interview component of Natsal. Participants who reported having had vaginal, anal, or oral sex in the past four weeks were asked, “On how many occasions in the last four weeks have you had sex with a (woman/man)?,” with the clarification: “This means vaginal intercourse, oral sex, anal sex.” The answers to each question were totalled, so that our measures of frequency include vaginal, oral, and anal sex occasions with opposite and same sex partners. Those who reported not having had sex in the past four weeks and those reporting never having had sex were coded as 0.

Preferred frequency of sex was derived from answers to the question, “Thinking of the way things are for you these days, which one of these would you really prefer?” which was asked of those reporting having had at least one opposite or same sex partner in the past year. The response options were: to have sex much more often than I do now; to have sex a bit more often; it is about right as it is; to have sex a little less often; to have sex much less often than I do now. This question was not asked in Natsal-1, so comparisons were only possible between Natsal-2 and Natsal-3.

Independent variables were derived from questions asked in the Natsal questionnaire, available from the study website (www.natsal.ac.uk ). Participants who reported having had a paid job of at least 10 hours a week in the past 10 years were asked details of this occupation, from which they were categorised into: higher managerial, administrative and professional occupations; intermediate; and routine and manual occupations. Participants who did not have a paid job of at least 10 hours a week in the past 10 years were coded as missing from this variable. The derivation of socioeconomic groups based on occupation was not possible with Natsal-1 data, so this variable was measured from only Natsal-2 and Natsal-3.

### Statistical analysis

We analysed data from men and women aged 16-44 years, the core age group that was included in all three Natsals, using Stata version 15.1 accounting for stratification, clustering, and weighting of the sample. Data were weighted to account for differential probabilities of selection to the survey and for non-response bias by age, sex, and region.

We present descriptive analyses (percentages and 95% confidence intervals) of trends across the three Natsals in actual and preferred frequency of sex by age group, marital status, and social class/employment status, separately for men and women. We examined frequency of sex using several different measures: median number of occasions of sex in the past month, the proportion of participants reporting no sex in the past month, the proportion reporting having sex 10 or more times in the past month, and the proportion reporting that they would like to have sex more often. We calculated odds ratios for each measure of frequency, comparing each Natsal survey to the previous one (ie, Natsal-2 *v* Natsal-1 and Natsal-3 *v* Natsal-2). We tested for interactions between Natsal survey measures and sociodemographic variables to examine whether the change in frequency of sex over time varied according to age group, marital status, and social class.

Using data from Natsal-3 only, we examined the factors associated with having sex four or more times in the past month (that is, more than the median number of occasions of sex for Natsal-3, identified in the descriptive analyses), using logistic regression analyses adjusted for age.

### Patient and public involvement statement

Members of the public were involved in the study at the pilot stage and as participants.

## Results

**Fig 1:**
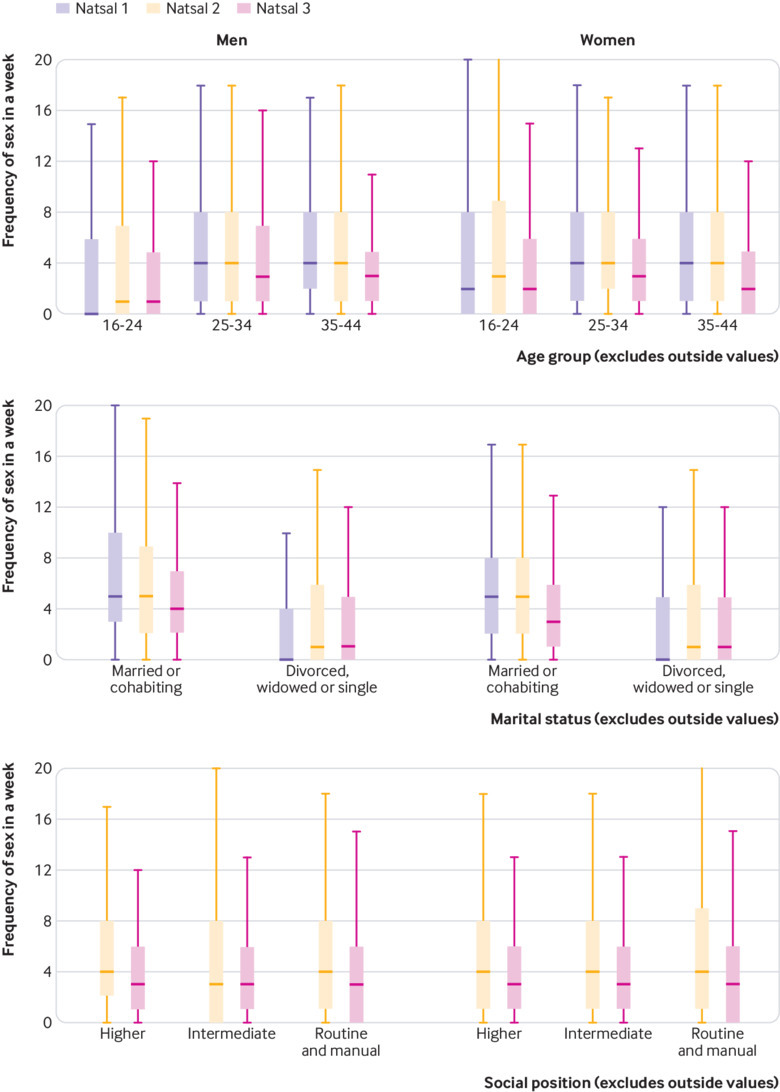
Median and interquartile range for frequency of sex in past four weeks by age, marital status, and socioeconomic position

Median number of occasions of sex in the past month was four in Natsal-1 and Natsal-2 and three in Natsal-3 among women; and three in all surveys among men (fig 1). The proportion reporting no sex in the past month fell from 28. 5% in Natsal-1 to 23.0% in Natsal-2 among women and from 30.9% to 26.0% among men, then increased significantly in Natsal-3 to 29.3% among women and 29.2% among men (table 1, table 2). The proportion reporting sex 10 or more times in the past month increased from 18.4% in Natsal-1 to 20.6% in Natsal-2 among women and from 19.9% to 20.2% among men, then fell in Natsal-3 to 13.2% among women and 14.4% among men.

**Table 1:** Actual and preferred frequency of sex: women aged 16-44

	Natsal-1	Natsal-2		Natsal-3		P for interaction
	% (95% CI)	% (95% CI)	OR 2 *v* 1	% (95% CI)	OR 3 *v* 2	
No sex last month						
Overall	28.5 (27.2 to 29.7)	23.0 (21.9 to 24.2)	0.75 (0.69 to 0.82)	29.3 (27.9 to 30.7)	1.38 (1.26 to 1.52)	
Age group*:						
16-24	42.3 (39.7 to 44.8)	35.2 (32.3 to 38.1)	0.74 (0.63 to 0.87)	38.8 (36.5 to 41.2)	1.17 (1.00 to 1.38)	0.005
25-34	22.3 (20.6 to 24.0)	16.9 (15.4 to 18.4)	0.71 (0.61 to 0.82)	23.2 (21.5 to 25.1)	1.49 (1.29 to 1.73)	
35-44	22.5 (20.8 to 24.4)	20.3 (18.7 to 21.9)	0.87 (0.75 to 1.01)	27.2 (24.6 to 29.9)	1.47 (1.24 to 1.74)	
Marital status:						
Married/cohabiting	13.2 (12.1 to 14.3)	9.2 (8.3 to 10.3)	0.67 (0.58 to 0.78)	15.1 (13.5 to 16.8)	1.74 (1.46 to 2.08)	<0.001
Single/divorced/widowed	56.2 (54.0 to 58.3)	45.6 (43.5 to 47.9)	0.66 (0.58 to 0.74)	46.3 (44.4 to 48.2)	1.03 (0.91 to 1.15)	
Social position:						
Professional/managerial		19.2 (17.0 to 21.7)		23.7 (20.7 to 27.0)	1.30 (1.03 to 1.64)	0.27
Intermediate		19.8 (18.1 to 21.6)		24.0 (21.6 to 26.7)	1.28 (1.07 to 1.53)	
Manual/routine		19.1 (17.5 to 20.8)		26.5 (24.4 to 28.6)	1.53 (1.31 to 1.78)	
Sex 10 or more times in last month						
Overall	18.4 (17.4 to 19.4)	20.6 (19.5 to 21.7)	1.15 (1.05 to 1.26)	13.2 (12.2 to 14.2)	0.59 (0.52 to 0.66)	
Age group*:						
16-24	19.4 (17.4 to 21.5)	25.0 (22.5 to 27.6)	1.38 (1.14 to 1.68)	17.0 (15.2 to 18.8)	0.61 (0.51 to 0.74)	0.02
25-34	19.7 (18.3 to 21.2)	21.7 (20.0 to 23.4)	1.13 (0.98 to 1.29)	14.5 (12.9 to 16.3)	0.62 (0.52 to 0.73)	
35-44	15.9 (14.5 to 17.4)	16.4 (14.7 to 18.1)	1.03 (0.87 to 1.22)	8.7 (7.0 to 10.7)	0.49 (0.38 to 0.63)	
Marital status:						
Married/cohabiting	21.3 (20.2 to 22.6)	22.7 (21.2 to 24.2)	1.08 (0.97 to 1.21)	13.0 (11.6 to 14.5)	0.51 (0.44 to 0.59)	<0.001
Single/divorced/widowed	13.0 (11.5 to 14.6)	17.1 (15.5 to 19.0)	1.39 (1.16 to 1.67)	13.4 (12.1 to 14.9)	0.75 (0.63 to 0.89)	
Social position:						
Professional/managerial		19.3 (16.7 to 22.2)		12.2 (9.9 to 14.9)	0.58 (0.43 to 0.78)	0.96
Intermediate		19.9 (17.9 to 22.0)		12.9 (11.0 to 15.1)	0.60 (0.48 to 0.75)	
Manual/routine		23.2 (21.4 to 25.2)		14.8 (13.2 to 16.6)	0.58 (0.48 to 0.68)	
Would prefer sex more often†						
Overall		39.1 (37.7 to 40.6)		50.6 (49.0 to 52.3)	1.55 (1.42 to 1.69)	
Age group*:						
16-24		34.9 (31.8 to 38.1)		45.5 (42.8 to 48.2)	1.59 (1.35 to 1.87)	0.56
25-34		43.7 (41.5 to 45.9)		53.7 (51.5 to 56.0)	1.47 (1.29 to 1.66)	
35-44		37.2 (34.8 to 39.6)		51.3 (48.0 to 54.6)	1.63 (1.38 to 1.91)	
Marital status:						
Married/cohabiting		38.0 (36.2 to 39.9)		51.0 (48.7 to 53.3)	1.68 (1.49 to 1.89)	0.02
Single/divorced/widowed		41.6 (39.0 to 44.1)		49.9 (47.6 to 52.2)	1.35 (1.19 to 1.54)	
Social position:						
Professional/managerial		47.3 (43.9 to 50.8)		57.1 (53.2 to 61.0)	1.44 (1.17 to 1.77)	0.25
Intermediate		40.7 (38.1 to 43.3)		56.4 (53.1 to 59.7)	1.77 (1.50 to 2.10)	
Manual/routine		37.0 (34.7 to 39.4)		48.6 (46.1 to 51.2)	1.53 (1.33 to 1.76)	

* all participants in age group irrespective of sexual experience. †in Natsal-3, this was asked only to those reporting at least one sexual partner in past year, so same restriction made for Natsal-2. OR=odds ratio. For denominators, see supplementary material.

**Table 2:** Actual and preferred frequency of sex: men aged 16-44

	Natsal-1	Natsal-2		Natsal-3		P for interaction
	% (95% CI)	% (95% CI)	OR 2 *v* 1	% (95% CI)	OR 3 *v* 2	
No sex last month						
Overall	30.9 (29.5 to 32.3)	26.0 (24.5 to 27.4)	0.79 (0.71 to 0.86)	29.2 (27.6 to 30.9)	1.18 (1.06 to 1.31)	
Age group*:						
16-24	50.3 (47.4 to 53.1)	43.0 (39.6 to 46.4)	0.75 (0.63 to 0.89)	43.4 (40.8 to 46.0)	1.02 (0.85 to 1.21)	0.01
25-34	23.4 (21.6 to 25.4)	19.5 (17.6 to 21.5)	0.79 (0.67 to 0.93)	23.1 (20.9 to 25.6)	1.25 (1.04 to 1.50)	
35-44	20.5 (18.8 to 22.5)	19.4 (17.6 to 21.3)	0.93 (0.79 to 1.09)	22.8 (19.9 to 25.9)	1.22 (0.99 to 1.51)	
Marital status:						
Married/cohabiting	11.5 (10.4 to 12.7)	9.1 (8.0 to 10.4)	0.78 (0.65 to 0.93)	12.8 (11.0 to 14.9)	1.46 (1.17 to 1.83)	<0.001
Single/divorced/widowed	57.4 (55.0 to 59.6)	47.1 (44.7 to 49.6)	0.66 (0.58 to 0.76)	47.1 (44.9 to 49.3)	1.00 (0.87 to 1.14)	
Social position:						
Professional/managerial		15.7 (13.6 to 18.1)		21.6 (18.3 to 25.4)	1.49 (1.14 to 1.94)	<0.001
Intermediate		30.6 (27.2 to 34.1)		22.9 (19.6 to 26.7)	0.68 (0.52 to 0.88)	
Manual/routine		21.7 (19.9 to 23.6)		26.9 (24.6 to 29.4)	1.33 (1.13 to 1.56)	
Sex 10 or more times in last month						
Overall	19.9 (18.8 to 21.1)	20.2 (18.9 to 21.6)	1.02 (0.92 to 1.14)	14.4 (13.2 to 15.6)	0.66 (0.58 to 0.75)	
Age group*:						
16-24	16.6 (14.6 to 18.8)	20.0 (17.6 to 22.7)	1.26 (1.02 to 1.56)	14.1 (12.4 to 16.1)	0.66 (0.53 to 0.82)	0.05
25-34	23.2 (21.4 to 25.2)	23.0 (20.9 to 25.2)	0.99 (0.84 to 1.16)	17.9 (15.9 to 20.0)	0.73 (0.61 to 0.87)	
35-44	19.3 (17.5 to 21.2)	17.7 (15.7 to 19.9)	0.90 (0.74 to 1.09)	11.2 (9.1 to 13.8)	0.59 (0.45 to 0.77)	
Marital status:						
Married/cohabiting	25.5 (23.9 to 27.2)	24.1 (22.2 to 26.2)	0.93 (0.81 to 1.07)	15.5 (13.6 to 17.5)	0.58 (0.48 to 0.69)	<0.001
Single/divorced/widowed	12.2 (10.9 to 13.7)	15.3 (13.6 to 17.0)	1.29 (1.07 to 1.56)	13.2 (11.8 to 14.8)	0.85 (0.70 to 1.02)	
Social position:						
Professional/managerial		20.9 (18.4 to 23.6)		14.3 (11.7 to 17.2)	0.63 (0.48 to 0.83)	0.56
Intermediate		19.1 (16.2 to 22.3)		15.5 (12.7 to 18.8)	0.78 (0.57 to 1.05)	
Manual/routine		22.8 (20.9 to 24.9)		16.2 (14.4 to 18.2)	0.65 (0.54 to 0.78)	
Would prefer sex more often†						
Overall		51.2 (49.5 to 52.8)		64.3 (62.4 to 66.1)	1.61 (1.45 to 1.78)	
Age group*:						
16-24		48.2 (44.5 to 51.8)		57.7 (54.6 to 60.8)	1.34 (1.12 to 1.61)	0.09
25-34		51.6 (48.9 to 54.3)		66.0 (63.2 to 68.8)	1.70 (1.44 to 1.99)	
35-44		52.7 (49.8 to 55.5)		67.3 (63.7 to 70.8)	1.75 (1.44 to 2.13)	
Marital status:						
Married/cohabiting		50.4 (8.2 to 52.7)		66.4 (63.8 to 68.9)	1.94 (1.68 to 2.24)	<0.001
Single/divorced/widowed		52.4 (49.8 to 55.0)		61.3 (58.7 to 63.8)	1.25 (1.09 to 1.44)	
Social position:						
Professional/managerial		55.5 (52.2 to 58.7)		70.8 (66.8 to 74.5)	1.90 (1.51 to 2.39)	0.18
Intermediate		57.8 (53.5 to 62.0)		67.9 (63.6 to 71.9)	1.38 (1.08 to 1.77)	
Manual/routine		46.1 (43.7 to 48.5)		61.1 (58.2 to 63.8)	1.70 (1.46 to 1.97)	

* all participants in age group irrespective of sexual experience. †in Natsal-3, this was asked only to those reporting at least one sexual partner in past year, so same restriction made for Natsal-2. OR=odds ratio. For denominators, see supplementary material.

Declines in frequency of sexual activity between Natsal-2 and Natsal-3 were evident across all age groups for women and for all but 16-24 year olds for men. They were largest among those aged 25 and over. Between these surveys, median number of occasions of sex in the past month fell from four to two among 35-44 year old women and from four to three among men in this age group. Prevalence of sexual inactivity in the past month was highest among participants aged under 25 in both surveys, but increased odds of sexual inactivity between Natsal-2 and Natsal-3 were higher among those aged 25-44. Sizeable declines in the odds of reporting occurrence of sex 10 or more times in the past month were seen across all age groups between Natsal-2 and Natsal-3. Among 35-44 year olds, the odds of doing so were halved among women and decreased markedly among men. (table 1, table 2).

Frequency of sexual activity was higher among participants who were currently married or cohabiting across all three surveys, but the decline over time was significantly greater in this group (table 1, table 2). Among married or cohabiting men and women, sexual inactivity in the past month was significantly higher in Natsal-3 than Natsal-2, whereas the odds of reporting frequency of 10 or more times a month were roughly halved. Declines of this magnitude between Natsal-2 and Natsal-3 were not seen among the currently single (P for interaction <0.001) so that the difference by relationship status narrowed. No significant variation was seen in any of the measures of sexual frequency by occupation.

**Fig 2:**
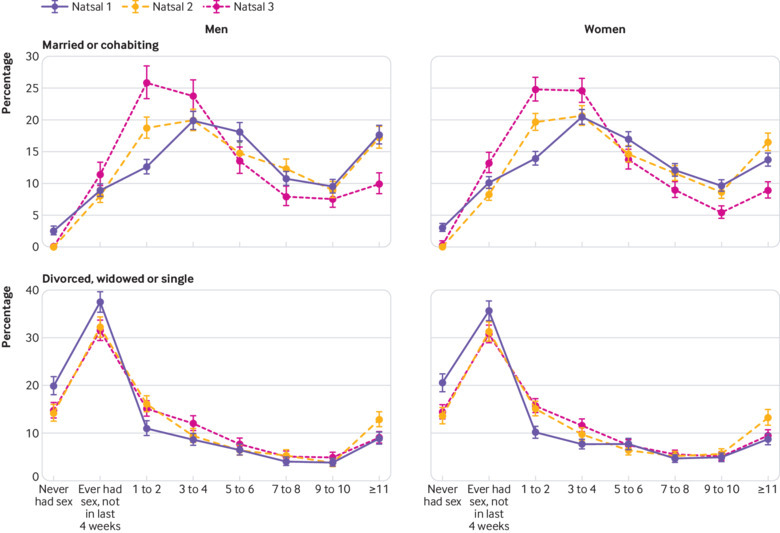
Ever sexual activity and sexual frequency in the past four weeks by Natsal survey

Figure 2—in which we distinguish between participants who have never had sex and those who have ever had sex but not in the past four weeks and display alongside frequency in the past four weeks—indicates that changes in sexual inactivity between surveys have contributed little to the observed decline in sexual frequency. The proportion of participants with no sexual experience fell between Natsal-1 and Natsal-2 but remained relatively stable between Natsal-2 and Natsal-3 among both married and single participants. Fig 2 indicates that the trend towards lower sexual frequency overall is largely attributable to the decline in frequency among sexually active married or cohabiting participants. The decline among those currently single is comparatively modest, aside from a reduction in the proportion having sex at frequencies greater than 10 times in the past four weeks between Natsal-2 and Natsal-3.

The proportion of participants expressing a preference for more frequent sex increased significantly between Natsal-2 (39.1% of women and 51.2% of men) and Natsal-3 (50.6% of women and 64.3% of men) and was higher among men than women. The increase was seen across all age groups and both marital status categories (table 1, table 2), with evidence of a steeper increase among those married or cohabiting (P values for interaction: 0.02 and <0.001).

Table 3 shows age adjusted associations between odds of having sex four or more times in the past month and selected variables in Natsal-3. Occurrence of sex at least once a week (that is, four or more times in the past month) was most strongly associated with being married or cohabiting (P<0.001); being in work or training (P<0.001); working longer rather than shorter hours (P<0.001); and with not having depression (P<0.001). Age adjusted odds for sex at least once a week decreased significantly with poorer health status and with increasing disability and increased with earnings, more markedly among men (test for trend: men: P<0.001; women P=0.02). They were higher among those living in households of two people as opposed to one (men: P<0.001; women: P=0.003), were significantly higher among men intending pregnancy (P<0.001), and almost twice as high among women intending pregnancy (P<0.001). Associations approaching significance were seen among men whose work was other than manual or routine (P=0.07) and who had children aged under five in the household (P=0.08) and among women who lived in rented accommodation (P=0.09). For men, an inverse association approaching significance was observed between recent masturbation and sexual activity; among women, the association was reversed and strongly significant, whereby recent masturbation was associated with higher odds of having sex at least once a week.

**Table 3:** Associations between sex four or more times in the past month and selected variables in Natsal-3

	Men				Women			
	% (95% CI)	Age adjusted OR	P value	N (unweighted/weighted)	% (95% CI)	Age adjusted OR	P value	N (unweighted/weighted)
Employment status:			<0.001				<0.001	
Full time education	25.1 (21.7 to 28.7)	0.32 (0.25 to 0.41)		872/652	32.3 (29.1 to 35.6)	0.50 (0.41 to 0.62)		1060/626
Employed/ training	46.0 (43.7 to 48.3)	1 (ref)		2561/2855	43.1 (41.1 to 45.1)	1 (ref)		3191/2386
Unemployed	35.5 (30.6 to 40.8)	0.59 (0.46 to 0.76)		444/360	38.0 (32.4 to 43.9)	0.74 (0.57 to 0.96)		387/243
Sickness/disability	32.1 (23.8 to 41.7)	0.56 (0.37 to 0.86)		133/113	34.7 (26.0 to 44.5)	0.72 (0.47 to 1.10)		142/102
Looking after home	41.8 (23.7 to 62.5)	0.88 (0.38 to 2.03)		35/ 44	42.5 (39.0 to 46.1)	0.96 (0.81 to 1.14)		1036/664
Retired	-	-		1/1	-	-		2/2
Social position*:			0.07				0.79	
Professional/managerial	46.5 (42.3 to 50.9)	1 (ref)		724/875	40.7 (36.9 to 44.5)	1 (ref)		856/688
Intermediate	46.6 (42.1 to 51.0)	0.97 (0.76 to 1.25)		656/703	43.1 (40.1 to 46.2)	1.06 (0.86 to 1.31)		1373/1029
Manual/routine	42.4 (39.8 to 45.1)	0.81 (0.66 to 1.00)		1890/1888	43.5 (41.2 to 45.7)	1.01 (0.84 to 1.23)		2392/1557
Hours worked per week:			<0.001				<0.001	
50 or more	47.0 (42.0 to 52.1)	1.02 (0.81 to 1.28)		494/583	39.4 (31.3 to 48.2)	0.79 (0.54 to 1.16)		183/146
35 to 49	46.9 (44.1 to 49.7)	1 (ref)		1712/1930	45.3 (42.4 to 48.2)	1 (ref)		1612/1177
10 to less than 35	39.1 (33.1 to 45.4)	0.69 (0.51 to 0.92)		383/364	41.5 (38.6 to 44.5)	0.87 (0.73 to 1.03)		1417/1062
Less than 10 hours	46.3 (24.6 to 69.5)	0.89 (0.33 to 2.41)		25/ 23	37.5 (27.5 to 48.6)	0.7 (0.44 to 1.13)		97/ 65
Not in paid work	29.1 (26.4 to 32.0)	0.42 (0.35 to 0.52)		1431/1122	37.1 (34.8 to 39.3)	0.68 (0.59 to 0.80)		2496/1560
Household income (£):			<0.001†				0.02†	
<2500	32.8 (23.2 to 44.2)	0.53 (0.32 to 0.89)		113/106	38.3 (30.3 to 47.1)	0.71 (0.48 to 1.04)		196/123
2500-4999	38.4 (29.0 to 48.9)	0.69 (0.44 to 1.10)		131/103	38.1 (31.5 to 45.1)	0.71 (0.51 to 0.99)		265/140
5000-9999	39.0 (31.0 to 47.8)	0.71 (0.48 to 1.05)		193/190	36.2 (31.6 to 41.1)	0.67 (0.51 to 0.86)		565/291
10 000-19 999	36.6 (31.7 to 41.8)	0.64 (0.49 to 0.84)		479/437	45.1 (41.3 to 49.1)	0.99 (0.79 to 1.24)		891/545
20 000-29 999	41.4 (36.4 to 46.5)	0.79 (0.61 to 1.03)		542/538	45.4 (41.3 to 49.6)	1.01 (0.81 to 1.27)		757/537
30 000-39 999	45.7 (40.6 to 50.9)	0.95 (0.72 to 1.24)		504/522	43.1 (38.8 to 47.6)	0.92 (0.72 to 1.18)		656/481
40 000-49 999	46.9 (41.3 to 52.6)	1.00 (0.76 to 1.32)		407/454	41.7 (36.8 to 46.8)	0.89 (0.69 to 1.15)		517/409
50 000+	46.9 (42.8 to 51.1)	1 (ref)		710/884	44.4 (40.5 to 48.4)	1 (ref)		804/712
Marital status:			<0.001				<0.001	
Married/cohabiting	50.2 (47.3 to 53.0)	1 (ref)		1542/2095	48.3 (46.1 to 50.5)	1 (ref)		2517/2190
Divorce/widowed	35.5 (26.9 to 45.1)	0.61 (0.40 to 0.93)		132/120	28.8 (23.5 to 34.8)	0.49 (0.37 to 0.65)		358/226
Single	31.3 (29.1 to 33.5)	0.33 (0.27 to 0.40)		2372/1809	32.1 (30.1 to 34.1)	0.36 (0.31 to 0.41)		2939/1604
Tenure:			0.22				0.09	
Own	41.6 (39.1 to 44.1)	1 (ref)		2024/2093	40.0 (37.9 to 42.2)	1 (ref)		2678/2047
Rented	41.7 (39.0 to 44.6)	1.03 (0.88 to 1.20)		1752/1724	42.3 (40.2 to 44.5)	1.08 (0.96 to 1.23)		2859/1805
Rent free	34 (27.5 to 41.2)	0.77 (0.56 to 1.06)		241/187	35.5 (29.3 to 42.2)	0.79 (0.59 to 1.07)		251/153
Household size:			<0.001				0.003	
One	34.7 (30.6 to 39.1)	1 (ref)		691/478	35.2 (31.2 to 39.5)	1 (ref)		656/312
Two	48.3 (44.7 to 52.0)	1.78 (1.40 to 2.25)		919/902	44.2 (41.2 to 47.2)	1.46 (1.17 to 1.81)		1473/914
Three or more	39.9 (37.6 to 42.3)	1.28 (1.03 to 1.59)		2428/2633	40.3 (38.5 to 42.2)	1.24 (1.02 to 1.51)		3687/2790
Child under 5 in household:			0.08				0.23	
No	40.1 (38.1 to 42.1)	1 (ref)		3338/3150	40.2 (38.3 to 42.0)	1 (ref)		4046/2907
Yes	45.1 (41.0 to 49.3)	1.19 (0.98 to 1.44)		715/883	42.2 (39.5 to 44.8)	1.09 (0.95 to 1.25)		1783/1123
Self rated health:			<0.001				0.007	
Very good	45.8 (43.1 to 48.5)	1 (ref)		1862/1851	41.8 (39.6 to 44.1)	1 (ref)		2618/1858
Good	38.2 (35.5 to 41.1)	0.72 (0.62 to 0.85)		1722/1731	41.2 (38.9 to 43.6)	0.98 (0.85 to 1.12)		2498/1691
Fair	33.6 (28.4 to 39.3)	0.58 (0.44 to 0.77)		398/387	36.7 (32.3 to 41.4)	0.81 (0.65 to 1.01)		579/390
Bad	33.9 (22.3 to 47.8)	0.57 (0.32 to 1.04)		71/ 64	26.7 (19.2 to 35.8)	0.51 (0.33 to 0.79)		134/ 91
Disability:			0.001				0.006	
None	42.7 (40.7 to 44.8)	1 (ref)		3300/3282	41.7 (40.0 to 43.4)	1 (ref)		4559/3134
Non-limiting	34.6 (29.2 to 40.5)	0.70 (0.53 to 0.91)		370/368	40.9 (36.3 to 45.6)	0.97 (0.79 to 1.19)		610/433
Limiting	34.4 (29.0 to 40.3)	0.68 (0.52 to 0.88)		382/382	34 (30.1 to 38.2)	0.72 (0.60 to 0.88)		659/462
Depressive symptoms‡			<0.001				<0.001	
No	44.3 (42.3 to 46.3)	1 (ref)		3473/3509	43.5 (41.9 to 45.2)	1 (ref)		4908/3427
Yes	27.1 (22.4 to 32.4)	0.47 (0.36 to 0.61)		405/376	33.0 (29.0 to 37.2)	0.63 (0.52 to 0.77)		697/451
Happy in relationship¶:			<0.001				<0.001	
Other	46.0 (41.3 to 50.8)	1 (ref)		604/714	45.2 (41.8 to 48.6)	1 (ref)		1107/858
Yes	58.0 (54.7 to 61.2)	1.67 (1.31 to 2.13)		1264/1534	55.0 (52.4 to 57.5)	1.45 (1.21 to 1.72)		1991/1537
Last masturbated:			0.09				<0.001	
Past 7 days	40.8 (38.5 to 43.1)	1 (ref)		2402/2276	49.7 (46.3 to 53.1)	1 (ref)		1166/791
Between 1 and 4 weeks	45.4 (41.2 to 49.8)	1.20 (0.98 to 1.47)		690/736	43.7 (40.2 to 47.3)	0.79 (0.65 to 0.96)		1109/766
Over 4 weeks	45.1 (41.0 to 49.3)	1.18 (0.98 to 1.43)		782/873	39.5 (37.6 to 41.4)	0.66 (0.56 to 0.77)		3307/2304
Currently trying to conceive:			<0.001				<0.001	
No	41.4 (39.5 to 43.4)	1 (ref)		3510/3464	41.4 (39.8 to 43.0)	1 (ref)		4968/3436
Yes	53.7 (47.6 to 59.7)	1.63 (1.26 to 2.12)		364/419	56.7 (50.8 to 62.4)	1.87 (1.47 to 2.39)		389/282

* Categorisation based on guidance from Office for National Statistics (https://www.ons.gov.uk/methodology/classificationsandstandards/otherclassifications/thenationalstatisticssocioeconomicclassificationnssecrebasedonsoc2010 ). †test for trend. ‡Participants were asked whether they had often been bothered by feeling down, depressed, or hopeless in the past two weeks and whether they had often been bothered by little interest or pleasure in doing things in the past two weeks, with a validated two question patient health questionnaire (PHQ-2). ¶Participants were asked to rate how happy they were in their relationship from 1 (very happy) to 7 (very unhappy); responses of 1 or 2 were regarded as denoting participants who were happy with their relationship. OR=odds ratio. Denominators vary across variables because of item non-response.

## Discussion

### Principal findings

Our data show that sexual frequency fell in Britain between Natsal-2 and Natsal-3. The most recent Natsal data show that fewer than half of men and women aged 16 to 44 have sex at least once a week. Those aged under 25 years and those currently single are less likely to be sexually active, but we saw the steepest declines in sexual frequency in those aged 25 and over and those married or cohabiting. At the same time, the proportion of men and women saying that they would prefer more frequent sex increased. Men and women in better physical and mental health report having sex more frequently, as do those who are fully employed and those with higher earnings.

### Strengths and limitations

The study benefits from drawing on a large, representative, population based sample. Limitations are nevertheless important to consider when interpreting the findings. Not all variables of interest were measured in all three surveys and some, use of pornography for example, were measured in none. The latest survey, Natsal-3, was completed six years ago, and findings from the fourth survey will not be available before 2024. Because the data are cross sectional, we cannot infer causality, and because they are based on self reports they are susceptible to response and reporting bias. Although we counted occasions of both opposite and same sex activities when calculating sexual frequency, we were unable to carry out analyses by sexual orientation of the participants. The majority of men and women who reported same sex activity had also experienced opposite sex activity, so the numbers reporting exclusively same sex activity were too small to carry out detailed comparative analyses.

### Comparison with other studies

A decline in sexual frequency has also been seen in Australia,^3^ Finland,^1^ Japan^4^ and the United States,^2^ although the studies show differences in which groups are most affected. Data from Australia, Finland, and the US are broadly consistent with our data, showing large declines in frequency among married people and those in early middle age. In the US, however, where the General Social Survey sample has no upper age limit, declines were largest among those in their 50s.^2^ In Finland, by contrast, middle aged men were the only participants to report a rise in frequency between 2007 and 2015.^1^ In Japan, sexual inactivity has been most marked among young, single people.^4^

Because patterns vary across settings, attempts to explain the trends must be specific to context. Several hypotheses can be considered in relation to our data. The decline in sexual frequency seems to coincide with two events of possible importance: the introduction of the iPhone in 2007 and the global recession of 2008. Others have suggested that increasing use of social media has resulted in increasing experience of “virtual” as opposed to real world sexual encounters, 22 23 and the media have sometimes linked increasing use of pornography with decreasing frequency of partnered sex. Use of pornography was not measured in Natsal to date, so we were unable to explore its association with sexual frequency, but in the US study,^2^ declines in sexual frequency were largest among those who did not watch pornography. The economic recession bears closer scrutiny. Although we found that men in full employment and those with higher earnings reported more frequent sex, the decline in frequency was seen among those in both higher and lower status jobs (but not among those in work of intermediate status), suggesting that different explanations are needed at each end of the socioeconomic spectrum.

Changing norms around sex may affect both reported and actual frequency. The social pressure to over-report sexual activity may have eased. Further, gender equality may now extend to the sexual sphere; where women might previously have felt obliged to meet their partner’s sexual needs irrespective of their own, they might now be less inclined to do so.^24^ Most compelling among the explanations, perhaps, given the age and marital status of the people most affected, relates to the stress and “busyness” of modern life, such that work, family life, and leisure are constantly juggled.^25^ Life in the digital age is considerably more complex than in previous eras, the boundary between the private space of home and the public world outside is blurred, and the internet offers considerable scope for diversion.^26^

### Conclusions and policy implications

Is the decline in sexual frequency a public health concern? Fears of an effect on fertility—prevalent in Japan where sharp declines in both sexual frequency and fertility rates have coincided—are not supported by our finding, or others’,^27^ of a higher frequency of sex where the intention is to conceive. The effect on health and wellbeing is less clear than it first seems.^28^ Research has shown no additional health benefits of a sexual frequency of more than once a week^29^ and indicates that any such benefits may depend not on sexual activity itself but on intimate contact^30^; holding hands or hugging has been shown to lower blood pressure and heart rate among women.^31^ A link between sexual frequency and relationship satisfaction and stability would have implications for public health, but here the evidence is equivocal.^32^ Caution is needed in interpreting the strong association between relationship satisfaction and sexual frequency because of the difficulty of determining causal direction, but others have shown that relationship satisfaction depends more on the quality than quantity of sex,^33^ particularly among women.^34^ Nevertheless, that close to half of women and almost two thirds of men report wanting to have sex more often merits concern, as does—notwithstanding the possibility of reverse causality—the strong relationship between lower sexual frequency and depression and self related health.

The wider implications of the decline in sexual frequency are perhaps more worrying. Should frequency of sexual contact serve as a barometer for more general human connectedness, then the decline might be signalling a disquieting trend. The decrease in sexual activity is interesting, unexplained, and warrants further exploration.

**What is already known on this topic**

A decline in the proportion of people who are sexually active and in the frequency of sex among those who are sexually active has been seen in several countries
Little is known about these trends in Britain, and the lifestyle factors associated with sexual frequency
There is evidence that regular sexual activity is beneficial to health and wellbeing

**What this study adds**

The decline in sexual frequency in Britain has been driven by a reduction in frequency among the sexually active, as opposed to an increase in the proportion who have never had sex
The decline in sexual frequency has occurred most notably among married or cohabiting people and among those in early middle age
Sexual frequency is higher among those in better mental and physical health and among those fully employed and financially better off

## Web extra

Extra material supplied by authors

**Supplementary table:** **Description of sample by Natsal survey**

## Notes

Contributors: KW contributed to the planning, conduct, and reporting of the work described in the article and is responsible for the overall content. As guarantor, she accepts full responsibility for the work and/or the conduct of the study, had access to the data, and controlled the decision to publish. The corresponding author attests that all listed authors meet authorship criteria and that no others meeting the criteria have been omitted. KW participated in the design and implementation of all three Natsal studies and prepared the first draft of the paper with contributions from MP, KM, and ES. MP carried out the statistical analysis with contributions from ES and KM. All authors contributed to data interpretation, reviewed successive drafts, and approved the final version of the report.

Ethical approval: Natsal-3 was approved by the Oxfordshire Research Ethics Committee A (reference: 09/0604/27). The University College Hospital and North Thames multi-centre research ethics committees and local research ethics committees in Britain approved Natsal-2.

Transparency declaration: As lead author I (KW) affirm that the manuscript is an honest, accurate, and transparent account of the study being reported; that no important aspects of the study have been omitted; and that any discrepancies from the study as planned (and, if relevant, registered) have been explained.

Data sharing: Data from the National Surveys of Sexual Attitudes and Lifestyles (Natsal) are available from: http://data-archive.ac.uk/
