# Supplementary material for: Changes in, and factors associated with, frequency of sex in Britain: evidence from three National Surveys of Sexual Attitudes and Lifestyles (Natsal)
Source: BMJ. 2019 May 7;365:l1525. doi: 10.1136/bmj.l1525 (PMC6503462; doi:10.1136/bmj.l1525)
Supplement: Supplementary file 1 — Supplementary table: Description of sample by Natsal survey [file welk047983.ww1.pdf]

	Natsal-1			Natsal-2			Natsal-3		
	%	95% CI	N (unweighted/ weighted)	%	95% CI	N (unweighted/ weighted)	%	95% CI	N (unweighted/ weighted)
**Response rate***	**66.8**			**65.4**			**57.7**		
**Sex**									
Male	50.5	49.6, 51.5	6000/6954	49.5	48.4, 50.6	4762/5525	50.0	48.8, 51.2	4060/4038
Female	49.5	48.6, 50.4	7765/6811	50.5	49.4, 51.6	6399/5636	50.0	48.8, 51.2	5842/4041
**Age group (years)**									
16 to 24	30.7	29.7, 31.7	3377/4224	27.3	26.2, 28.4	2673/3044	30.3	29.2, 31.3	3869/2445
25 to 34	36.9	36.0, 37.9	5641/5080	35.5	34.5, 36.5	4320/3958	34.1	33.0, 35.2	4012/2755
35 to 44	32.4	31.5, 33.4	4747/4461	37.3	36.2, 38.3	4168/4159	35.6	34.4, 37.0	2021/2880
**Marital status**									
Married/cohabiting	61.2	60.0, 62.3	8248/8413	59.1	58.0, 60.3	5729/6584	53.3	52.0, 54.5	4066/4294
Divorced/widowed/single	38.9	37.7, 40.0	5510/5345	40.9	39.7, 42.0	5401/4551	46.7	45.5, 48.0	5812/3765
**Social position**									
Professional/managerial				21.8	20.8, 22.8	2137/2131	23.2	22.0, 24.4	1583/1565
Intermediate				27.6	26.7, 28.6	2932/2695	25.7	24.6, 26.9	2031/1733
Manual/routine				50.6	49.3, 51.9	4723/4941	51.1	49.7, 52.5	4289/3450

Supplementary table 1: Description of sample by Natsal survey (restricted to ages 16-44 years).

***Response rate for entire sample (Natsal-1: 16-59 years; Natsal-2: 16-44 years; Natsal-3:16-74 years).**

Denominators vary across variables because of item non-response.
